# Identification of Adrenomedullin-Induced S-Nitrosylated Proteins in JEG-3 Placental Cells

**DOI:** 10.1007/s43032-021-00663-7

**Published:** 2021-08-30

**Authors:** Yingting Li, Liuying Zhong, Cheuk-Lun Lee, Philip C.N. Chiu, Min Chen

**Affiliations:** 1 Department of Obstetrics and Gynecology, Department of Fetal Medicine and Prenatal Diagnosis, Key Laboratory for Major Obstetric Diseases of Guangdong Province, The Third Affiliated Hospital of Guangzhou Medical University, 63 Duobao Road, Liwan District, Guangzhou, China; 2 The University of Hong Kong Shenzhen Key Laboratory of Fertility Regulation, The University of Hong Kong-Shenzhen Hospital, Shenzhen, China; 3 Department of Obstetrics and Gynaecology, LKS Faculty of Medicine, The University of Hong Kong, Hong Kong, China

**Keywords:** ADM-induced S-nitrosylated proteins, ANX II, Adrenomedullin, Human extravillous cytotrophoblast, Invasion

## Abstract

Extravillous cytotrophoblast (EVCT) is responsible for trophoblast invasion, which is important during placentation. Dysregulation of the process leads to pregnancy complications. S-nitrosylation of proteins is associated with cell invasion in many cell types. Adrenomedullin (ADM), a polypeptide expressed abundantly in the first-trimester placentas, induces EVCT invasion by upregulation of protein S-nitrosylation. This study aimed to identify the S-nitrosylated proteins induced by ADM in the JEG-3 placental cells. By using affinity chromatography followed by mass spectrometric analysis, tubulin, enolase, eukaryotic translation initiation factor 4A1, actin, annexin II (ANX II), and glyceraldehyde 3-phosphate dehydrogenaseprotein-1 were found to be S-nitrosylated by ADM. In vitro treatment with ADM or S-Nitrosoglutathione (GSNO) significantly increased the ANX II surface expression, but not its total expression in the JEG-3 cells. Translocation of ANX II to cell surface has been reported to act as a cell surface receptor to plasmin, plasminogen, and tissue plasminogen activator (tPA), thereby stimulating cell invasion and migration. However, in this study, ADM-induced surface expression of ANX II in the JEG-3 cells was not associated with changes in the secretory and membrane-bound tPA activities. Future studies are required to understand the roles of surface expression of S-nitrosylated ANX II on trophoblast functions. To conclude, this study provided evidences that ADM regulated the nitric oxide signaling pathway and modulated trophoblast invasion.

**Supplementary Information:**

The online version contains supplementary material available at 10.1007/s43032-021-00663-7.

## Introduction

During placental development, human trophoblasts differentiate along two cell lineages leading to the formation of extravillous cytotrophoblasts (EVCT) and villous cytotrophoblasts [1]. EVCT are responsible for trophoblast invasion, a key process for successful placentation. They produce matrix metalloproteinase and urokinase plasminogen activator that degrade the extracellular matrix of decidua for the invasion process [2]. In contrast to tumor invasion, EVCT invasion is controlled temporally and is spatially restricted to the inner third of the myometrium [3]. Both the uterus and the placenta produce inhibitory and stimulatory factors to fine-tune the invasion process [4]. Dysregulation of the process is associated with a wide range of pregnancy complications, including intrauterine growth restriction, preeclampsia, and choriocarcinoma [1]. However, the mechanisms controlling human EVCT invasion are poorly understood.

S-nitrosylation involves covalent attachment of a nitric oxide (NO) group to a cysteine thiol side chain. Protein S-nitrosylation can either activate or inactivate protein function, depending on the protein nitosylated. S-nitrosylation of proteins has been associated with invasion in many cell types. In the human lung epithelial cell line, Beas-2B, stabilization of B-cell lymphoma (Bcl)-2 protein through S-nitrosylation leads to malignant transformation and increase in cell invasiveness [5]. In the MCF-7 breast cancer cells, β-estradiol induces NO production which activates c-V-src sarcoma viral oncogene homolog (Src) tyrosine kinase through S-nitrosylation of the cysteine-498 residue. This process reduces E-cadherin expression in the MCF-7 cells followed by disruption of cell-cell adhesion and activation of cell invasion [6].

In our previous study [7], adrenomedullin (ADM) is shown to induce EVCT invasion by upregulation of protein S-nitrosylation, indicating that protein S-nitrosylation plays an important role in EVCT functions. ADM also increases the total protein S-nitrosylation levels in EVCT [7]. The objective of this study was to identify the S-nitrosylated proteins in the JEG-3 placental cells induced by ADM.

## Materials and Methods

### Cell Culture and Cell Lines

JEG-3 is a choriocarcinoma cell line (HTB-36™, ATCC, USA) derived from human placenta. It shows EVCT characteristics including expression of human leukocyte antigen G, and is frequently employed as an EVCT model in studies on trophoblast invasion and migration in vitro [8, 9]. The cells were authenticated and tested for contamination. The JEG-3 cells were cultured in DMEM-F12 (Sigma) medium supplemented with 10% FBS (Sigma) in standard cell culture condition at 37°C in an atmosphere of 5% CO_2_ in air. The cells at passages 5–20 and with over 90% viability as determined by trypan blue exclusion test were used in this study.

### Sodium Dodecyl Sulfate-Polyacrylamide Gel Electrophoresis

Protein samples were mixed with 5X sample loading buffer (0.5 M Tris-HCl, 2% SDS (w/v), 0.02% bromophenol blue (v/v), 5% β-mercaptoethanol (v/v) and 10% glycerol (v/v), pH 6.8) and denatured for 10 min at 95°C. The denatured proteins were then resolved in 10% polyacrylamide gel. Sodium dodecyl sulfate-polyacrylamide gel electrophoresis (SDS-PAGE) was performed in a Mini-protein 3 System (Bio-Rad) with 25 mA current at room temperature until the dye front reached the gel bottom. The protein bands were visualized by a silver staining kit (GE Healthcare) according to the manufacturer’s instructions.

### Western Blotting

SDS-PAGE resolved proteins were blotted on a polyvinylidene fluoride (PVDF) membrane (Millipore, Billerica, MA, USA) using a wet tank transfer protocol. Afterward, the PVDF membrane was treated with 0.5% BSA in PBS for 30 min at room temperature. The membrane was incubated successively with diluted primary antibody at 4°C overnight and appropriate horse radish peroxidase (HRP)-conjugated secondary antibody for 60 min at room temperature. In between the incubation procedures, the blots were washed for 5 times (10 min each) with PBS containing 0.05% Tween 20 (PBST). Finally, the protein bands were visualized on an autoradiography film using enhanced the chemiliminescence (ECL) reagent (Santa Cruz, CA, USA) and were quantified using the ImageJ software (National Institutes of Health, Bethesda, Maryland, USA).

### Purification of S-Nitrosylated Proteins

S-nitrosylated proteins were purified by the S-Nitrosylated Protein Detection Assay Kit (Cayman, Ann Arbor, MI, USA) and streptavidin-conjugated agarose (Merck). The assay employs a biotin switch method to biotinylate the S-nitrosylated proteins. Briefly, the free thiol groups of cell lysates (20×10^6^ cells) were blocked by the blocking reagent of the kit. The S-nitrosothiols of proteins were then reduced and covalently labeled with maleimide-biotin. The biotin-labeled proteins were resuspended in 500 μL PBS and were purified by incubation with 25 μL of streptavidin-conjugated agarose at 4°C for 3 h. The purified S-nitrosylated proteins were resolved by SDS-PAGE and were visualized by silver staining or western blot as described above. Biotin conjugated myelin basic protein (ab792; Abcam) was added as a loading control of the protein purification process and was detected by polyclonal antibody against myelin basic protein (ab28541; Abcam).

### Invasion Assay of Human Primary Trophoblast

The involvement of human subjects in this study was approved by the Institutional Review Board of The University of Hong Kong/Hospital Authority Hong Kong West Cluster. Human placental tissue was collected from the termination of pregnancy in the first 12 weeks of gestation performed in the Department of Obstetrics and Gynaecology, The University of Hong Kong. All samples were processed immediately after collection. The primary trophoblast was isolated by our published protocols [10, 11]. The trophoblast invasion was quantified by a transwell invasion assay (Corning, NY). In brief, primary human trophoblasts (5 × 10^5^) in serum-free DMEM with/without supplementation of 10 nM ADM or 100 μM GSNO were allowed to invade through a basement membrane for 24 h. The invaded cells on the membrane were stained with 0.1% crystal violet and quantified by measuring the absorbance at 595 nm after dissolving the crystal violet dye from the membrane by 10% acetic acid.

### Mass Spectrometry Analysis of S-Nitrosylated Proteins After ADM Treatment

JEG-3 cells (2.5×10^5^) were treated with 10 nM ADM for 24 h at 37°C in an atmosphere of 5% CO_2_ in air. The dosages of ADM used were physiological and effective according to our pervious study [7]. Cells treated with PBS were used as a control. S-nitrosylated proteins were purified, resolved by SDS-PAGE, and visualized by silver staining. The S-nitrosylated protein bands in SDS-PAGE were excised and digested in situ with trypsin (0.1 mg/ml in 25 mM NH_4_HCO_3_, pH 8.0). The digested peptides were reduced by 1,4-dithiotreitol, alkylated by iodoacetamide, recovered with Millipore C18 ZipTips, and dissolved in 60% acetonitrile-0.1% TFA containing α–cyano-4-hydroxycinammic acid matrix. The peptide-matrix samples were then analyzed with the matrix-assisted laser desorption/ionization time-of-flight mass spectrometry (MALDI-TOF-MS/MS, Centre for PanorOmic Sciences, The University of Hong Kong) to obtain the peptide mass spectra (MDS Sciex, South San Francisco, CA), which were compared with the protein sequences in the public protein databases at the Swiss-Prot (http://www.ebi.ac.uk/swissprot/).

### Effect of ADM and GSNO on S-Nitrosylated Annexin II Level

JEG-3 cells (2.5×10^5^) were treated with 10 nM ADM or 100 μM S-Nitrosoglutathione (GSNO), an endogenous nitrogen oxide, for 24 h. The concentrations of ADM and GSNO used were based on our previous studies indicating their biological effects in the in vitro study [7]. Cells treated with PBS were used as a control. S-nitrosylated proteins were purified and resolved by SDS-PAGE. S-nitrosylated annexin II (ANX II) was detected by western blot using a polyclonal anti-ANX II antibody (1:1000; ab41803; Abcam).

### Effect of ADM and GSNO on Cell Surface Expression of ANX II

ANX II can act as a cell surface receptor for tissue plasminogen activator (tPA) [12]. The ANX II-tPA interaction upregulates the tPA-dependent conversion of plasminogen into active plasmin, and thereby stimulates cell invasion and migration [13]. Therefore, we hypothesized that S-nitrosylation may enhance the cell surface expression of ANX II on JEG-3 cells. S-nitrosylation has also been demonstrated to increase cell surface receptor expression in other cell types [14]. To test this hypothesis, the JEG-3 cells were treated with 10 nM ADM or 100 μM GSNO for 24 h at 37°C in an atmosphere of 5% CO_2_ in the air. Cells treated with PBS were used as a control. Cell surface ANX II expression was then determined by flow cytometric analysis using 1 μg/mL polyclonal antibodies against ANX II (ab41803; Abcam). In brief, 0.5×10^6^ JEG-3 cells after treatment were washed with blocking buffer (1% BSA and 0.1% sodium azide in PBS) followed by incubation with anti-ANX II antibody at 4°C for 3 h. The cells were then washed with PBS and resuspended in 500 μL of blocking buffer containing 1 μg/mL Alexa Fluor-488 secondary antibody (Invitrogen). After 1 h of incubation at 4°C in dark, the cells were washed and analyzed by a flow cytometer (BD FACSCantoII Analyzer, BD Biosciences) equipped with a 488 nm argon laser. Fluorescence signals were measured using a 525 nm band pass filter. The positive threshold level was set according to the background fluorescence of isotypic control. The data were analyzed by the WinMDI software (The Scripps Research Institute Cytometry Software, San Diego, CA, USA).

The surface expression of ANX II of ADM/GSNO-treated JEG-3 cells was confirmed by immunostaining. The JEG-3 were seeded in 24-well plates (5 × 10^4^ cells/well) and cultured for 24 h. The cells were then treated with ADM/GSNO as described above, washed with PBS, and fixed in 4% paraformaldehyde for 30 min. After washing, the cells were incubated with a 1 μg/mL antibody against ANX II (ab41803; Abcam) overnight at 4°C. The bound antibodies were detected by an Alexa Fluor-555-conjugated secondary antibody (diluted 1:1000; Invitrogen). Isotypic antibodies were used as control.

### Effect of ADM on tPA Activity

JEG-3 cells (1 × 10^4^) in 100 μL culture medium were treated with 10 nM ADM for 24 h at 37°C in an atmosphere of 5% CO_2_ in air. Cells treated with PBS were used as a control. The enzyme activities of secretory tPA in conditioned media or membrane-bound tPA on the JEG-3 cells after treatment were quantified by the Sensolyte AFC tPA activity assay kit (AnaSpec, Fremont, CA, USA) according to the manufacturer’s instructions. The kit measures the ability of tPA in digesting a synthetic substrate 7-amino-4-trifluoromethylcoumarin (AFC). Briefly, conditioned media (40 μL) or 1 × 10^4^ JEG-3 cells were incubated with 50 μL of diluted substrate solution at 37°C for an hour. The amount of digested AFC was then quantified fluorometrically by an ELISA plate reader with an excitation wavelength of 380 nm and an emission wavelength of 505 nm. Treatment with 20 μM specific tPA inhibitor provided by the kit was included in the assay as a negative control. The tPA activity was expressed as percentage of fluorescence intensity relative to the control.

### Data Analysis

All values were expressed as mean ± standard error of mean (SEM). The data were analyzed by the SigmaStat 2.03 software (Jandel Scientific, San Rafael, CA, USA). For all experiments, the nonparametric rank sum test for comparisons was used to identify differences between groups. If the data were normally distributed, parametric Student *t* test was used as the post test. *P* < 0.05 was considered statistically significant.

## Results

### ADM and GSNO Increases Trophoblast Invasion

**Fig. 1:**
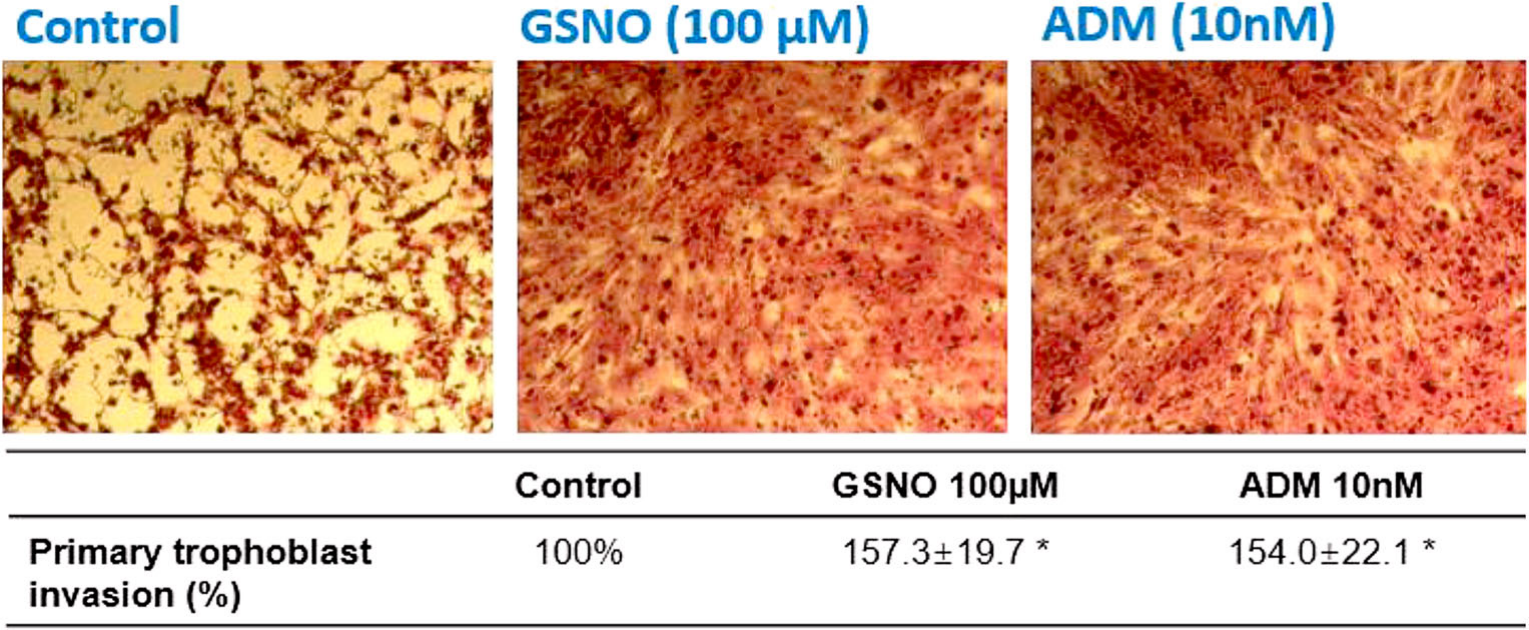
Effect of ADM and GSNO on trophoblast invasion. Effects of different concentrations of ADM and GSNO on invasion of human primary trophoblast (*N=*4). *P<0.05 when compared with the control without treatment

Both ADM and GSNO treatments significantly (*P* < 0.05) enhanced invasiveness of human primary trophoblast by 154.0 ± 22.1% and 157.3 ± 19.7%, respectively, when compared with the control (Fig. 1), consistent with our previous study [7].

### Identification of ADM-Induced S-Nitrosylated Proteins

**Fig. 2:**
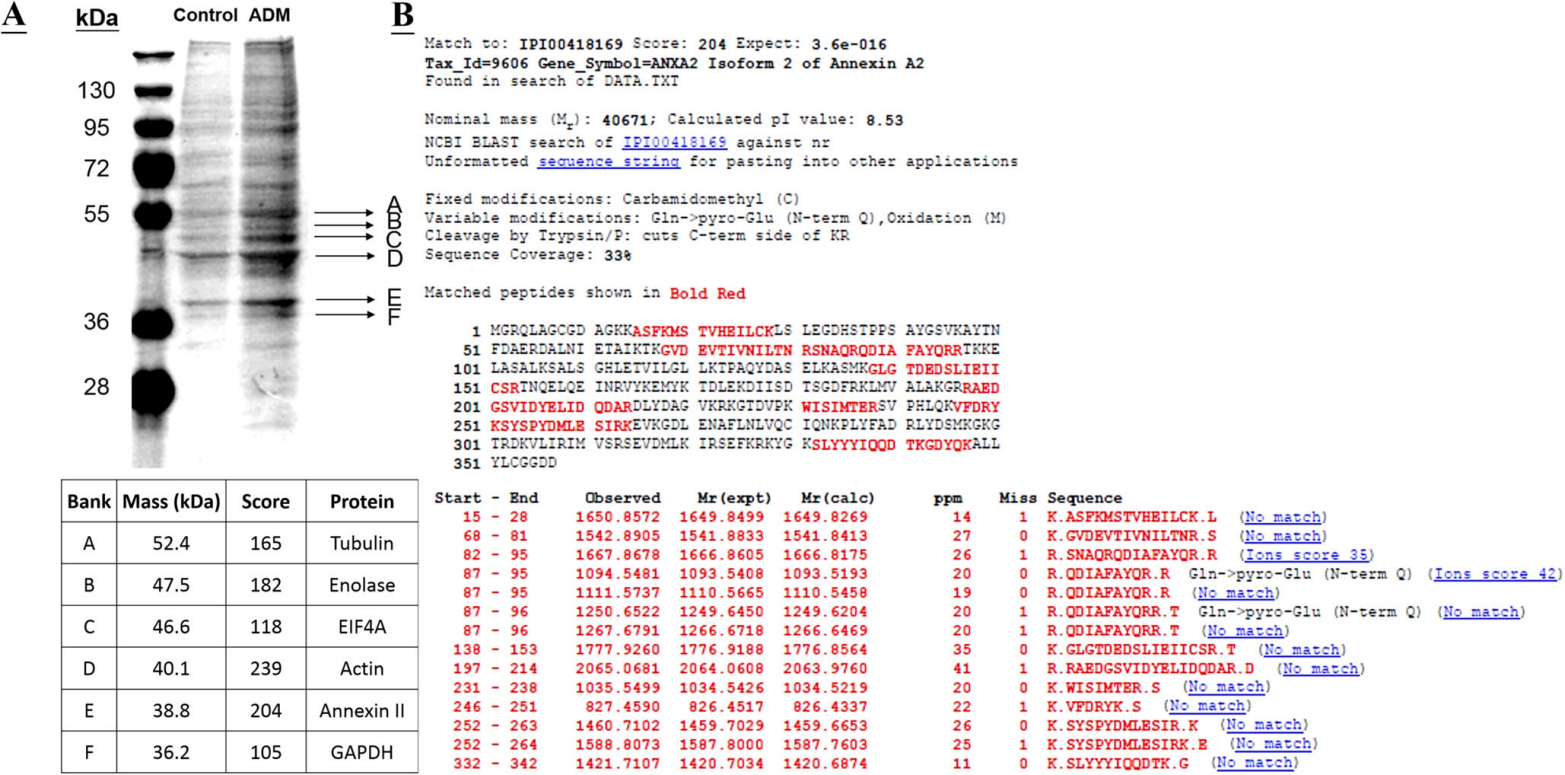
Identification of ADM-induced S-nitrosylated proteins by SDS-PAGE and mass spectrometry analysis. A—Protein S-nitrosylation after ADM treatment was analyzed by SDS-PAGE followed by silver staining; mass spectrometry results showing the protein identities and Mascot scores for the corresponding bands. The results shown are representative of two replicate experiments; B—identification of ANX II by MALDI-TOF-MS/MS. The peptide sequences were compared with that in the public protein (Homo sapiens) databases at the NCBInr database. The protein sequence matched was shown in bold red which had total sequence coverage of 33%

ADM treatment increased the total S-nitrosylated protein level in the JEG-3 cells (Fig. 2A). Six protein bands were extracted from the silver-stained gel and subjected to peptide mass fingerprinting by mass spectrometric analysis (Fig. 2A). Database search identified tubulin, enolase, eukaryotic translational initiations factor 4A1, actin, ANX II (Fig. 2B), and glyceraldehyde 3-phosphate dehydrogenase protein-1 as the ADM-induced S-nitrosylated proteins with Mascot protein score higher than 100. In this report, ANXII was investigated for its expression in the JEG-3 cells.

### ADM Increases S-Nitrosylated ANX II Level in JEG-3 Cells

**Fig. 3:**
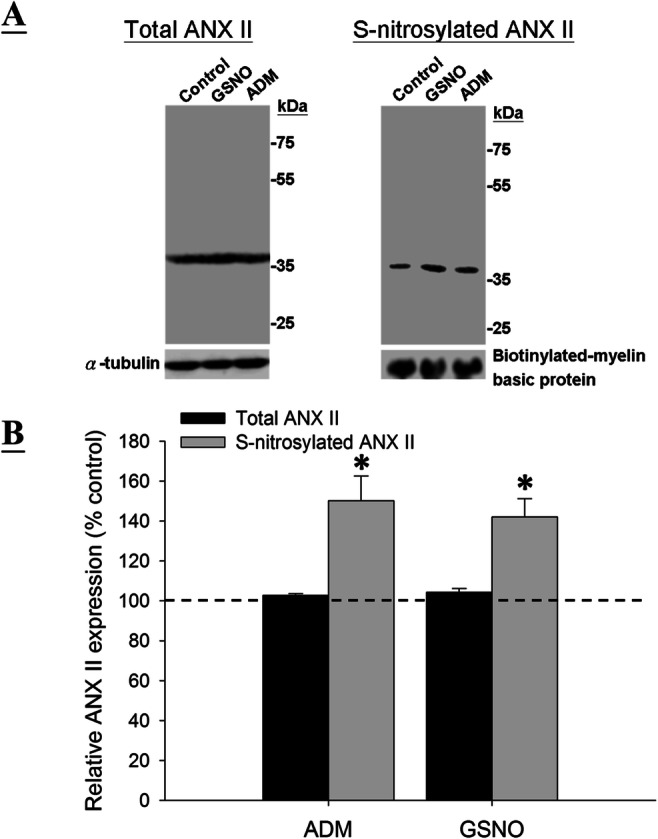
Effect of ADM and GSNO on S-nitrosylated ANX II level in JEG-3 cells. A—Western blot analysis of total and S-nitrosylated ANX II using 1:1000 anti-ANX II antibody; B—semi-quantification of total and S-nitrosylated ANX II by densitometry (*N* = 3). All values are presented as percentage changes relative to the control without treatment. *P<0.05 when compared with the control

The ADM-induced ANX II S-nitrosylation in the JEG-3 cells was confirmed by Western blotting (Fig. 3 and Supplementary Fig. S1). While ADM had no effect on the total ANX II expression (Fig. 3 and Supplementary Fig. S1), it significantly (*P* < 0.05) increased the S-nitrosylated ANX II level in the JEG-3 cells (Fig. 3B). Similar observation was obtained with GSNO treatment, indicating that ADM-induced NO upregulation may be responsible for the induction of S-nitrosylated ANX II (Fig. 3 and Supplementary Fig. S1).

### S-Nitrosylation Increases the Cell Surface ANX II Expression on JEG-3 Cells

**Fig. 4:**
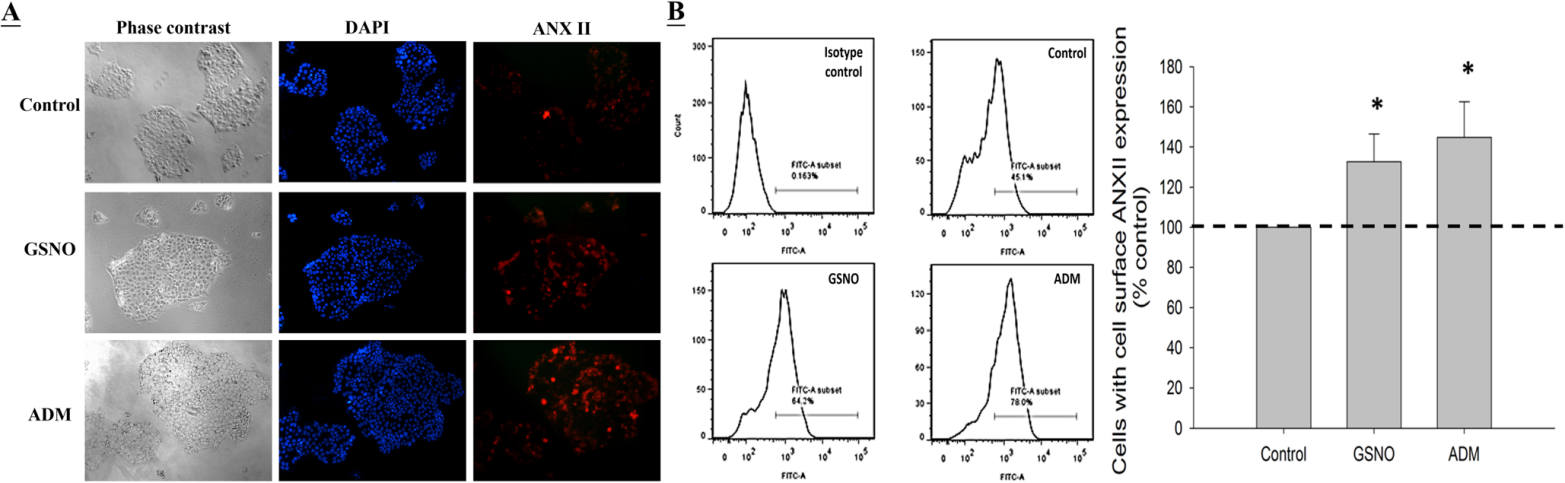
Effect of ADM and GSNO on cell surface ANX II expression level in EVCT. A—Immunostaining of JEG-3 cells using 1 μg/mL anti-ANX II antibody followed by Alexa Fluor-555-labeled secondary antibody. Results shown are representative of three replicated experiments. B—Left: flow cytometry analysis of cell surface ANX II expression using 1 μg/mL anti-ANX II antibody. The results shown are representative of five replicate experiments; right: quantitative determination of cell surface ANX II expression (*N* = 5). *P<0.05 when compared with the control without treatment

Immunostaining (Fig. 4A) and flow cytometric analysis (Fig. 4B) demonstrated that both ADM and the endogenous nitrogen oxide GSNO treatment significantly (*P* < 0.05) enhanced the cell surface ANX II expression on JEG-3 cells when compared with the control. ADM and GSNO increased the cell surface ANX II expression by 44.8 ± 17.7% and 32.6 ± 13.9% respectively (Fig. 4B).

### ADM Has no Effect on tPA Activity

**Fig. 5:**
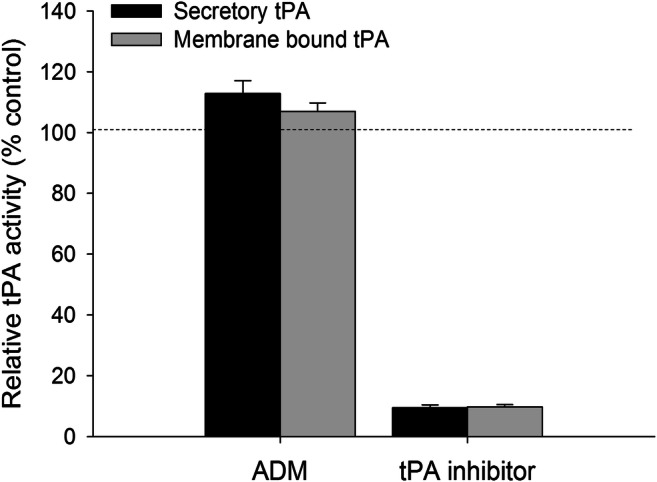
Quantitative analysis of secretory and membrane-bound tPA activity of JEG-3 cells using tPA activity assay kit. All values are presented as percentage changes relative to the control without treatment (*N* = 5)

Cell surface ANX II upregulates the tPA activity and stimulates cell invasion in endothelial cells [13]. Since ADM-induced S-nitrosylation increased the surface expression of ANX II, we tested the tPA activity of JEG-3 cells. Our results showed that ADM treatment did not affect both the secretory and membrane-bound tPA activity in the JEG-3 cells (Fig. 5). In contrast, tPA inhibitor significantly (*P* < 0.05) suppressed tPA activity.

## Discussion

In the first trimester of pregnancy, EVCT are differentiated from cytotrophoblasts of the placental villa. The EVCT invade the decidua and remodel the spiral artery, which transforms the spiral arteries to low-resistance and high-flow vessels ensuring sufficient fetal-maternal exchange. Failure in trophoblast invasion causes the “Great Obstetrical Syndromes,” a spectrum of pregnancy complications such as fetal growth restriction (FGR), preterm labor, and late spontaneous miscarriage [15]. Data of this study suggested that ADM-induced S-nitrosylation of ANX II and possibly other proteins in EVCT may take part in the regulation of EVCT invasion during early pregnancy.

Both ADM and the GSNO treatment enhanced the invasion of EVCT and surface ANX II expression. ANX II is a calcium-binding cytoskeletal protein belonging to the annexin family [13]. Increased ANX II expression has been reported in a number of cancers, such as breast [12], liver [16], and lung [17]. Studies have shown that ANX II stimulates cancer invasion [18]. In trophoblasts, ANX II is involved in syncytiotrophoblast maturation and differentiation [19], but its role in trophoblast invasion is still not clear. Our studies provide a possible link between s-nitrosylation, ANX II, and EVCT invasion [7]. Interestingly, a study on nitroso-proteomes of human placentas showed that ANX II is one of the proteins with decreased S-nitrosylation level in pre-eclamptic placentas [20], suggesting ANX II S-nitrosylation may be involved in mediating trophoblast invasion and spiral artery remodeling. The above observations together with our mass spectrometry results suggest that ANX II can be a potential target for ADM-induced protein S-nitrosylation to regulate ADM-induced EVCT invasion.

ADM enhances human trophoblast invasion via increasing urokinase plasminogen activator expression and activity [7]. ADM-induced ANX II may also play a role in cell invasion by regulating the plasminogen/plasmin system. ANX II can act as a cell surface receptor to bind plasmin, plasminogen, and tPA [12]. This binding stimulates the tPA-dependent conversion of plasminogen into active plasmin by bringing plasminogen and tPA into close spatial proximity. The catalytic efficiency for human recombinant tPA to generate plasmin was significantly increased when bound to purified ANX II [21]. Upregulation of plasminogen and plasmin activity facilitates degradation of extracellular matrix and matrix metalloproteinase (MMP) activation, leading to enhanced cell invasion and migration [22]. In the MDA-MB231 breast cancer cells, silencing of ANX II gene abolishes tPA binding, resulting in inhibition of tPA-dependent plasmin generation and suppressed cell motility, demonstrating the importance of ANX II in enhancing cell motility through upregulation of tPA-dependent plasmin generation [23]. tPA has been detected in human trophoblast [24]. Although poor tPA response and impaired plasmin-dependent proteolysis have been associated with early recurrent abortion in pregnant women [24], the functional role of tPA on trophoblast invasion is unknown.

To function as a receptor that promotes plasmin formation, ANX II has to be translocated to the cell surface. In this study, we demonstrated that S-nitrosylation may be a factor regulating the translocation of ANX II to the cell surface. ANX II can exist as a monomer or a heterotetramer, which consists of two S100 calcium binding protein A10 (p11) protein subunits and two ANX II monomer subunits [19]. In the intracellular environment, ANX II exists as a monomer, while ANX II heterotetramer is localized to the cell plasma membrane [19]. Binding of p11 subunit to ANX II appears to direct the protein to the cell surface [22]. Translocation of ANX II monomers from the cytoplasm to the plasma membrane is regulated by factors such as tyrosine phosphorylation [25] and interaction with heat shock protein 60 (HSP60) [26]. In endothelial cells, heat stress leads to the translocation of ANX II to the plasma membrane in a p11 protein and tyrosine phosphorylation dependent manner [27].

Our results unexpectedly failed to find an effect of ADM on tPA activity. Apart from acting as a cell surface receptor for tPA, ANX II also participates in other cellular functions, such as regulation of cytoskeleton organization [28], DNA replication [29], and exocytosis [30]. Therefore, other mechanisms may be involved in modulating ADM-induced EVCT invasion. For example, ANX II has been colocalized with other binding proteins such as tenascin C and cathepsin B resulting in activation of extracellular matrix (ECM) degradation [18]. Tenascin C is a large extracellular matrix molecule in tumors and plays a crucial role in tumorigenesis [31]. In macrophages, ANX II-tenascin C interaction induces cell migration [32]. Overexpression of tenascin C stimulates migration of cancer cells [31]. Cathepsin B is a lysosomal cysteine protease that binds to ANX II and is detected on the cell surface of tumor cells [33]. In human colorectal cancer (CRC) cells, pro-cathepsin B interacts with the ANX II heterotetramer on the cell surface [34]. In endothelial cells, colocalization of ANX II heterotetramer and cathepsin B is involved in ECM degradation [35]. Cathepsin B expression in human trophoblast is positively correlated with the trophoblast invasiveness and preeclampsia [36]. Therefore, ADM-induced ANX II S-nitrosylation and surface expression may increase the binding of ANX II to cathepsin B and/or tenascin C to enhance EVCT cell invasiveness. ANX II is also important in actin remodeling and regulating cytoskeleton structures essential for cell migration [29].

While this study had mainly focused on ANX II, five other proteins were identified to have increased S-nitrosylation after ADM treatment. Of these proteins, tubulin and actin have been commonly reported to play roles in cell invasion and motility. The main function of tubulin is formation of microtubules, which is the essential element for cytoskeleton [37]. The ability of tubulin to polymerize and depolymerize from microtubules through addition and deletion of subunits at polymer ends provides the dynamics for cell motility [38]. Tubulin is involved in cell motility in epithelial cells and fibroblasts [37]. Actin is another important component of the cytoskeleton involved in cell motility [39]. In trophoblasts, actin organization has been associated with cell adhesion, fusion, and migration [40]. Epidermal growth factor (EGF) stimulates migration of trophoblast through actin cytoskeleton reorganization [41].

Enolase is a well-known glycolytic enzyme [42]. It has other functions, such as acting as a heat shock protein or plasminogen receptor [43, 44]. While there is no report on its role in trophoblast invasion, enolase may participate in cancer cell invasion. Enolase is expressed on the surface of invasive breast [45], lung [46], and pancreatic tumors [42]. In hepatocellular cancer, enolase expression is positively correlated with cell invasion [47]. S-nitrosylation of tubulin, actin, and enolase has been reported [48], but their possible roles in trophoblast invasion are unknown. S-nitrosylation of actin and enolase is decreased in pre-eclampsia [49] consistent with their possible involvement in trophoblast invasion.

Overall, this studies not only provide insights on the role of ADM in S-nitrosylation of EVCT but also raise the intriguing possibility of S-nitrosylation of ANX II and other surface proteins in mediating EVCT invasion. The study also highlights the potential use of ADM level or ANX II S-nitrosylation in trophoblast as early detection biomarker and/or therapeutic target of pregnancy complications associated with abnormal trophoblast invasion. Future studies are required to understand how S-nitrosylation of these proteins can modulate the spiral artery remodeling process activities of EVCT.

## Supplementary Information

ESM 1 (PDF 58 kb)

## Data Availability

Research data could be seen within the manuscript.
