# Supplementary material for: Identification of Adrenomedullin-Induced S-Nitrosylated Proteins in JEG-3 Placental Cells
Source: Reprod Sci. 2021 Aug 30;29(4):1296–304. doi: 10.1007/s43032-021-00663-7 (PMC8907116; doi:10.1007/s43032-021-00663-7)

Supplementary Fig. S1. Effect of ADM and GSNO on S-nitrosylated ANX II level in JEG-3 cells. Western blot analysis of total and S-nitrosylated ANX II using 1:1000 anti-ANX II antibody (N=3).

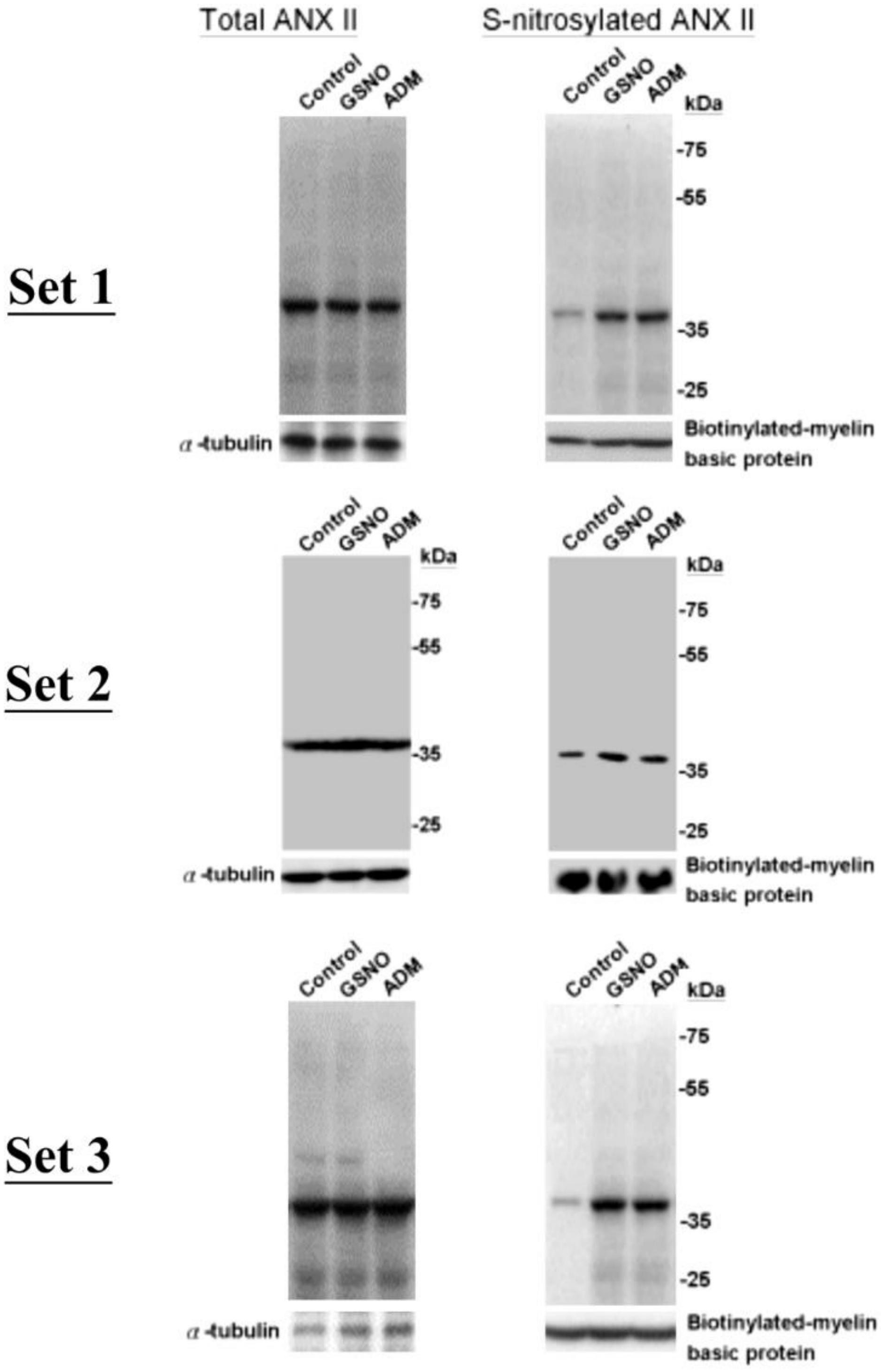

Supplement: Supplementary file 1 — (PDF 58 kb) [file 43032_2021_663_MOESM1_ESM.pdf]
